# Physiotherapy in the primary care oncology team

**DOI:** 10.4102/safp.v68i1.6231

**Published:** 2026-03-02

**Authors:** Lomé Prinsloo

**Affiliations:** 1 Private Practice, Cape Town, South Africa

**Keywords:** physiotherapy, cancer rehabilitation, lymphoedema, exercise, cancer-related fatigue, chemotherapy-induced peripheral neuropathy, prevalence, oncology care

## Abstract

As the incidence of cancer rises, medical advancements have made it possible to have increased survival rates among cancer patients. However, many experience persistent treatment-related side effects that negatively affect their quality of life. At the first international Oncology Physiotherapy Conference in 2018, it was agreed that undergraduate physiotherapy training provides a strong foundation for oncology rehabilitation. However, management of specific side effects such as cancer-related fatigue (CRF), chemotherapy-induced peripheral neuropathy (CIPN) and lymphoedema, necessitates additional oncology-specific knowledge and skills. Cancer-related fatigue is one of the most common and distressing symptoms during cancer treatment. Evidence supports the effectiveness of non-pharmacological interventions, such as exercise, to manage CRF, while pharmacological options remain limited. Referral to physiotherapy should thus be considered as a primary approach. Research also supports the use of physiotherapeutic interventions such as exercise and sensorimotor integration in the treatment of CIPN. Physiotherapists upskilled as lymphoedema therapists can deliver Complex Decongestive Therapy (CDT), the gold standard in treating lymphoedema. The Lymphoedema Association of South Africa (LAOSA) promotes standardised care through a 135-h certification process. Exercise for cancer patients should be promoted by all members of the multidisciplinary team, and referral to professionals such as physiotherapists should occur early in the treatment journey. This article aims to sensitise primary healthcare providers to the integral role physiotherapists play in the management of patients with cancer who experience CRF, CIPN and lymphoedema.

## Introduction

Globally, cancer is considered a leading cause of disease burden^1^ and mortality.^2^ However, because of increased screening efforts, improvements in technology and advances in treatments, the survival rates of patients with cancer have risen.^1,2,3^ Unfortunately, many cancer survivors suffer from cancer treatment-related side effects or impairments that negatively impact their quality of life.^2,4^ Physiotherapy is considered one of the main services provided by the oncology rehabilitation team.^5^ Physiotherapy interventions can play a role in the amelioration of some of these physical impairments,^6^ and should thus be considered as an integral part of the multidisciplinary approach to treating patients with cancer.

The National Cancer Policy Forum in the United States called for better integration of rehabilitation services into cancer care, including physiotherapy. They believe that by integrating these rehabilitation services, ideally from the start of the cancer diagnosis, long-term treatment-related disability and side effects may be reduced.^6^

Similarly, the Clinical Oncology Society of Australia (COSA) issued a guideline on exercise in cancer care. They recommended that exercise should be viewed as an adjunct therapy to mitigate the side effects of cancer and its treatments, and that it should be embedded as standard practice in cancer care. The promotion of physical activity and adherence to exercise guidelines should be encouraged by all members of the multidisciplinary team. They also suggest that best practice involves referring patients to professionals such as physiotherapists with experience in cancer care.^6,7^ There is a paucity of data on best practices for delivering physiotherapy and exercise in cancer care within the South African context. In 2018, the first international conference on physiotherapy in oncology was held in Amsterdam. Drawing 280 physiotherapists from 30 countries, this event served as a platform to showcase scientific advances and foster global collaboration in oncology rehabilitation.^6^ It was agreed that entry-level physiotherapy graduates are ideally suited to rehabilitate patients with cancer by playing a role in identifying and managing common cancer-related impairments such as pain, limited range of motion and reduced physical fitness. However, managing side effects such as lymphoedema, chemotherapy-induced peripheral neuropathy (CIPN) and cancer-related fatigue (CRF) requires further oncology-specific knowledge and advanced skills.^6^

## Physiotherapy in managing three common yet complex cancer-related side effects

### Cancer-related fatigue

Across the literature, CRF is noted to be one of the most common symptoms experienced by patients with cancer.^8,9,10,11^ It is described as distressing and has shown to negatively impact the quality of life of patients with cancer.^8,9,11,12^ The National Comprehensive Cancer Network (NCCN) defines CRF as a distressing and persistent sense of tiredness that may be physical, emotional or cognitive in nature. It is related to cancer or its treatment, is not proportional to recent activity and interferes with usual functioning.^8,9,11,14^ It may persist years after the cessation of treatment,^8,13^ with 20% – 30% of cancer survivors reporting ongoing and persistent fatigue 5–10 years after treatments have ended.^13^ Regardless of its high prevalence, it is still undertreated by clinicians and under-reported by patients.^13^ It is important to differentiate between fatigue and asthenia. Fatigue is directly correlated to mental or physical exertion, whereas asthenia is tiredness or exhaustion in the absence of exertion. Yet, CRF may not enjoy the attention it deserves, as fatigue is considered a normal symptom in oncology.^12^ A systematic review and meta-analysis conducted by Al Maqbali et al.^9^ in 2021 investigated the prevalence of fatigue in patients with cancer. They reported that 49% of patients with cancer experience fatigue.^9^ Therefore, it can be assumed that nearly half of patients with cancer encountered in clinical practice may be experiencing fatigue. These patients should ideally be screened and adequately referred for physiotherapy.

An evidence-based resource tool from the Oncology Nursing Society states that, to date, there are no evidence-based pharmacological agents or nutritional supplements for the treatment of CRF, apart from dexamethasone and erythropoietin, both of which could have detrimental side effects.^14^ However, evidence has shown exercise and other non-pharmacological therapies to be effective in reducing CRF.^14^ The Putting Evidence into Practice (PEP®) tool states that exercise and physical activity are considered the first-line management strategies in the treatment of CRF.^14^ As noted by Hilfiker et al.,^14^ other interventions with moderate to large effect sizes in reducing CRF while undergoing cancer treatments were: relaxation, massage, cognitive-behavioural therapy (CBT) combined with physical activity, as well as aerobic and resistance training. Yoga showed a large effect size for the reduction of CRF after cancer treatments.^14^ As many of these non-pharmacological interventions fall within physiotherapists’ scope of practice, referral to physiotherapy should be considered a primary approach or first-line therapy in the treatment of CRF.

Another study investigated the impact of a multimodal exercise and functional rehabilitation programme for managing CRF in patients with cancer. The multidisciplinary team, which included physiotherapists, achieved improvements in fatigue, functional ability, pain and quality of life among oncology patients.^12^ Additionally, physiotherapy should be viewed as a non-pharmacological treatment for fatigue in palliative patients living with advanced cancer.^11^ A study found that a physiotherapy programme, which included active exercises, myofascial release and proprioceptive neuromuscular facilitation techniques, significantly reduced the severity of fatigue in palliative patients.^11^

A practical recommendation for clinicians encountering a patient with CRF would be to follow the American College of Sports Medicine (ACSM) guidelines. The guideline recommends that cancer survivors should avoid inactivity and participate in moderate aerobic exercise (talk but not sing) for up to 30 min, 3 times a week, and 2 sessions a week of resistance training for 20 to 30 min.^15^ Patients living with cancer should be encouraged to avoid inactivity and be as physically active as their condition allows. Assessment includes asking the patient about the frequency of their aerobic and resistance training habits per week to ascertain whether they already meet the ACSM guidelines. The clinician must further consider if the patient would be safe exercising without medical supervision. If the answer is yes, the clinician advises on the guidelines. If the answer is no, the clinician advises on the recommended guidelines and refers the patient to an appropriate healthcare professional, such as a physiotherapist with experience in cancer care. Clinicians must therefore Assess, Advise and Refer.^15^

### Chemotherapy-induced peripheral neuropathy

The use of chemotherapy as an anti-cancer treatment is contributing to the increasing number of cancer survivors. This powerful medicine acts by targeting rapidly dividing cancer cells; however, healthy cells and tissues are not selectively spared. This often results in undesirable and negative side effects, which may impair the patient’s ability to complete their oncology treatment regimen, potentially reducing patient survival.^2,16^ Symptoms of CIPN include sensory disturbances in the hands and feet, such as numbness, tingling, pain and cold sensitivity. Motor impairments include cramping, reduced fine motor control and disturbances in balance and gait.^17^ The sequelae of CIPN negatively impact patients’ quality of life,^16^ and it is considered one of the most common and severe side effects of this treatment.^18^ Chemotherapy-induced peripheral neuropathy is mainly a sensory neuropathy, but it may affect the motor and autonomic systems.^2^ Chemotherapy-induced neural damage may be explained by different underlying pathophysiology mechanisms, which include Deoxyribonucleic acid (DNA), mitochondrial and myelin sheath damage, microtubule disruption, neuroinflammation, oxidative stress and altered ion channel activity.^2^

Chemotherapy-induced peripheral neuropathy is more likely to develop after intravenous infusion of epothilones, taxanes (especially paclitaxel and docetaxel), platinum-based agents (especially oxaliplatin or cisplatin) or vinca alkaloids.^2^ Evidence supports the use of some physiotherapeutic interventions in the management of CIPN. Some of the most widely used physiotherapeutic interventions in the management of CIPN include manual therapies such as massage, and treatment modalities such as photo biomodulation, electrotherapy, transcutaneous electrical nerve stimulation, neuro-developmental and cardiopulmonary therapies.^2^

A South African study investigated the correlation between exercise prescription, motor function and health-related quality of life in patients with CIPN. Exercise was associated with lowering the symptoms of CIPN and improvements in quality of life and physical well-being.^3^ Another randomised controlled trial investigated the preventative effect of neuromuscular training, including sensorimotor (SMT) and whole-body vibration (WBV) training, on CIPN. It focused on oxaliplatin and vinca alkaloids, among the most commonly used chemotherapy agents, with a high CIPN incidence of 70% – 90%. Patients were divided into three groups. The first interventional group received SMT, the second WBV training, and the control group received standard care. Sensorimotor training included balance exercises performed on progressively unstable surfaces, and WBV training utilised a vibrational platform. Both SMT and WBV training reduced the onset of CIPN in 50% – 70% of patients, compared to standard care. This effect was pronounced in patients receiving vinca alkaloids, compared to those receiving oxaliplatin. While both interventional groups reduced the onset of CIPN, SMT showed the most additional benefits in improving pain, burning sensation, vibrational sensitivity and lower leg strength. The SMT led to less chemotherapy dose reductions, lower mortality, improved physical activity and quality of life. The WBV training reduced CIPN and improved balance in a bipedal stance. Beyond prevention, these training programmes showed improvements in CIPN symptoms, potentially impacting patient mortality and adherence to treatment.^16^ A systematic review by Seth et al. found that resistance and endurance training, along with neural mobilisation, reduced CIPN symptoms and pain, and improved strength and quality of life.^2^

Addressing CIPN is therefore imperative as it may prevent chemotherapy dosage alterations or discontinuation, which could lead to better survival outcomes. It may also support greater participation in exercise, thereby enhancing patient quality of life, and aligns with global initiatives to integrate exercise into cancer care.

### Lymphoedema

Lymphoedema is a chronic and progressive condition where an abnormal collection of protein-rich fluid in the tissues below the skin occurs because of dysfunction in the lymphatic system, which could lead to severe swelling of the affected limb or body part. Primary lymphoedema is because of a developmental deficiency, and secondary lymphoedema is acquired because of the disruption of normal lymphatic channels.^19,20^ Predisposing factors for secondary lymphoedema include cancer-related treatments such as extensive lymph node dissections, radiotherapy and chemotherapy.^21^ It most often affects the arms or legs, but can involve other areas like the face, trunk or genitals. This condition is incurable and requires ongoing management. Early intervention improves management outcomes.^19,20^ The Lymphoedema Association of South Africa (LAOSA) has released a position statement defining lymphoedema, outlining its stages, discussing the standard of treatment and promoting its recognition as a Prescribed Minimum Benefit (PMB).^19^

Physiotherapists are among the healthcare practitioners eligible for lymphoedema certification training. Complex Decongestive Therapy (CDT) is regarded as the gold standard for the treatment of lymphoedema. This includes a collection of treatments such as manual lymph drainage, compression therapy (including multilayered lymphoedema bandaging and compression garments), exercise, skin care and home care. Techniques aimed at reducing the volume fall under the initial (intensive) phase. The use of garments is commonly used in the long-term management of lymphoedema, known as the maintenance phase.^19,20^ The LAOSA advocates for standardised care, which must be delivered by appropriately trained healthcare professionals who hold a 135-h certification. They manage the National Practice Register that lists certified lymphoedema therapists in South Africa. To find a practitioner, visit their website: www.laosa.co.za.^19^

## Conclusion

Medical advances have increased cancer survival rates, meaning patients are living longer with their cancer diagnoses. Dealing with the long-term sequelae of treatment-related adverse effects poses a challenging reality and may negatively affect an individual’s ability to thrive, even long after cancer treatments have ceased. Physiotherapists can, and should, play a vital role within the multidisciplinary team by helping to reduce these symptoms. Entry-level physiotherapists can effectively manage common problems like pain, limited range of motion and decreased physical fitness. More complex symptoms such as CRF, CIPN and lymphoedema require advanced physiotherapy skills and knowledge of oncology treatments. Physiotherapy is ideally suited to improve functional ability and enhance the quality of life for individuals with cancer, including those currently undergoing cancer treatments, those in remission and those receiving palliative care. Implementation of the proposed physiotherapy roles must be considered within the realities of the South African public health system, where allied health capacity is often limited. Approaches such as early integration of oncology training within physiotherapy undergraduate programmes, clinician upskilling, promoting awareness among medical clinicians, structured referral pathways and remote support may help extend the reach of physiotherapy in oncology care and warrant further investigation.
